# Collective motions and specific effectors: a statistical mechanics perspective on biological regulation

**DOI:** 10.1186/1471-2164-11-S1-S2

**Published:** 2010-02-10

**Authors:** Alessandro Giuliani

**Affiliations:** 1 Environment and Health Department, Istituto Superiore di Sanità, Viale Regina Elena 299, 00161 Roma, Italy

## Abstract

**Background:**

The interaction of a multiplicity of scales in both time and space is a fundamental feature of biological systems. The complementation of macroscopic (entire organism) and microscopic (molecular biology) views with a mesoscopic level of analysis able to connect the different planes of investigation is urgently needed. This will allow to both obtain a general frame of reference for rationalizing the burden of data coming from high throughput technologies and to derive effective operational views on biological systems.

**Results:**

The network paradigm in which microscopic level elements (nodes) are each other related by functional links so giving rise to both global (entire network) and local (specific) behavior is a promising metaphor to try and develop a statistical mechanics inspired approach for biological systems. Here we show the application of this paradigm to different systems going from yeast metabolism to murine macrophages response to immune stimulation.

**Conclusions:**

The need to complement the purely molecular view with mesoscopic approaches is evident in all the studied examples that in turn demonstrate the untenability of the simple ergodic approach dominant in molecular biology in which the data coming from huge ensemble of cells are considered as relative to a single ‘average’ cell.

## Background

The classical form in which biological systems are described (being they metabolic charts, gene expression regulation pathways, protein-protein interaction maps, food webs and so forth) corresponds to a set of nodes linked by edges in which the nodes are the basic elements of the described system (genes, proteins, metabolites and so forth) and the edges connecting them some rules of the kind ‘is transformed into’ or ‘is increased by’ [1,2].

With the development of high throughput methodologies these graphs became larger and larger and asked for some form of global analysis in order to get rid of their wild multiplicity.

The approach considering the graph as a system of differential equations in which an entering stimulus, correspondent to a modification of a peripheral node of the network, is progressively processed according to the wiring architecture and kinetics constraints of the network itself, while being the most potentially exhaustive avenue of research is severely hampered, in the case of biological systems, by a lot of problems. First of all the practical impossibility to attach to the whole set of edges reliable kinetic-like weights for quantifying the entity of the between elements correlation. Only in the case of very small networks this can be done by means of the statistical estimation of the parameters from experimental data, but it is well known that in physiological settings these weights can vary of orders of magnitude [3]. Moreover in many cases we cannot rely on the complete knowledge of the wiring diagram of the network.

For these (and other) reasons many authors preferred a purely topological approach to the analysis of biological networks, considering the presence of a link between two nodes as a pure yes/no binary relation and limiting themselves to statistical descriptions making use of the so called graph-invariants, i.e. a collection of indexes that, relying on the simple count of nodes and edges, enable the analyst to identify crucial elements of the network (like the so-called hubs, nodes engaged in a very large amount of relations) or to highlight specific features of the entire network architecture responsible for some aspects of the studied system behavior (this is the case of the so called ‘scale-free’ architecture that was demonstrated to be at the basis of the huge resilience of biological systems) [4-6].

The consideration of biological systems at the coarse-grain level of the graph topological approach is, in my opinion, a very important first step for the development of a sort of biological statistical mechanics in which the actual behavior of the global system can be predicted by a convenient statistics over its constituent parts.

In the case of statistical mechanics of inanimate systems this was the case with the Boltzmann microscopic definition of entropy as a statistical index computed over the microstates frequency distribution of the studied system [7]. This deliberate coarse-grain approach that abandoned the dream of following the trajectories of the single elements for a population level view, enabled scientists to get a link between microscopic and macroscopic physical descriptions [7-9].

 In the following we will try and describe the search for a Boltzmann-like approach to biology by the critical analysis of different regulation network-like systems.

## Results

### i) Essential by location: the case of yeast metabolic network

From a purely topological point of view, each node of a network is uniquely defined by its position in the graph. Obviously, when dealing with experimentally derived and not abstract networks, each node has a name (a particular gene, protein, metabolite) pointing to a rich basin of knowledge and evoking cognition resonance to the specialist mind and the same is true for the edges. However, if we are interested in discovering what can be inferred solely from topological information (so acquiring a Boltzmann-like statistical attitude sacrificing the unique personality of the element to the search of a mesoscopic principle), we should try and predict some relevant features of the studied system without relying on the particular ‘nature’ of nodes and edges, but only taking into consideration their connectivity pattern. In other terms all the properties relative to each node (edge) must be derived only by its pattern of relations and thus by its peculiar location in the complete graph. We checked for the possibility to derive, from purely topological information on the metabolic network of yeast (*Saccharomyces Cerevisiae*), the lethal character of genetic mutations [10]. The metabolic network of microorganisms is very well understood: it can be considered as a graph having enzymatic reactions as edges and metabolites as nodes. Since an enzymatic reaction is catalysed by one or more enzymes, an edge (or arc) can also represent the enzymes involved in the reaction. This opens the way to a straightforward analysis of the possibility to derive biologically meaningful features at a macroscopic scale (entire organism) from network topology: the elimination of an enzyme by a knock-out experiment implies the elimination from the network of the edge (or edges since the same enzyme can catalyze different reactions) corresponding to that particular enzyme [10,11]. If it is possible to pick up a connectivity descriptor able to unequivocally define essential enzymes (those enzymes whose lack provoke the yeast death) we can safely assume the biological relevance of the metabolism ‘wiring structure’, irrespective of the specific nature of the involved enzymes, and consequently deriving a mesoscopic biological principle.

**Figure 1:**
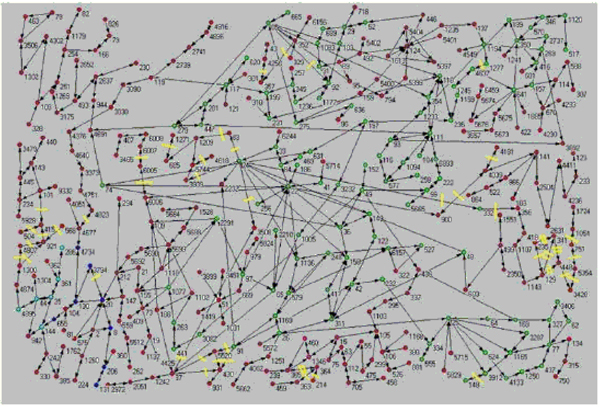
**The metabolic network of yeast is depicted in the figure, the enzymatic reactions correspond to edges, while the nodes point to the metabolites. **The yellow signs indicate the analyzed mutations.

In the considered case of yeast metabolic network, the analysis of 36 lethal mutations out of the 412 relative to enzymes involved in metabolism, reported in the Stanford repository (http://www-sequence.stanford.edu/group/yeast_deletion_project/deletions3.html) and in Jeong and colleagues [12], allowed us to discover that all of the enzymes corresponding to lethal mutations, when deleted, prevent the connections between the separate nodes [10]. No alternative path is available to connect the separate nodes and this explains the essential character of the mutation on a pure topological basis (Figure 1).

This ‘essentiality-by-location’ mesoscopic principle equating the lethal character of a mutation to the lack of an alternative path in the network, was confirmed by another study by our group [11] demonstrating that a double mutation involving two enzymes that *per se* are not essential acquires essentiality and then causes the death of the organism, if the double knock-out provokes the ‘lack of alternative path’ condition. This illustrates the emergent character of the ‘essentiality by location’ principle: the arising of lethality by the summation of two non lethal events derives from the existence of a global metabolism architecture and thus cannot be inferred by going in depth into the nature of the two enzymes, in other words is a collective emergent property of the network system [11].

It is worth noting we did not find any exception to this rule: if an alternative pathway does exist then the mutation is not lethal. These data suggest the lack of ‘purely kinetic’ lethal mutations, i.e. situations in which the poor kinetic properties of alternative paths do not allow the yeast to survive. This points to a remarkable difference between metabolic and artificial networks: if we think of a road map, an accident causing a block of a huge highway (kinetically optimal path) causes the traffic flow to shift into much narrower alternative roads (kinetically non-optimal paths), this will provoke soon or later a traffic jam that will make impossible a normal traffic flux with a consequent detrimental condition for the entire system. The fact we never observed such a situation allows for the speculation that kinetic constraints in biological networks are not hard-wired in the network architecture and can be relatively easily circumvented. There are in fact some experimental data demonstrating the possibility of many orders of magnitudes variations of kinetic parameters of biochemical reactions [3,13].

All in all this case showed us the existence of a mesoscopic level (the network) whose behavior cannot be simply derived by the knowledge of the constituting elements while, in an apparently counterintuitive manner with respect to the reductionist paradigm, influencing the microscopic level. In the subsequent paragraphs we will look for other examples of the need to seriously approach such mesoscopic organization [8].

### ii) Gene waves: gene function independent transcriptome motions

The almost totality of microarray experiments is aimed to find ‘gene expression signatures’ of different biological macrostates as specific pathologies or phenotypic consequences of mutations. The aim of these studies is to look for specific genes whose level of expression is affected by the cellular macrostate (e.g. tumor phenotype) so to give both a mechanistic explanation and some potentially useful diagnostic or therapeutical hints. Two implicit assumptions are hidden at the basis of these studies: 1) The existence of few ‘master genes’ that drive the observed phenotype while the great majority of gene products are completely unaffected by the studied condition and 2) A fully ergodic assumption of complete independence of the cells in the culture so that each observed variation in gene expression can be traced back to something happening inside an ‘average cell’ and thus explained in terms of ‘nodes-and-arrows’ regulation pathways involving intracellular molecular constituents [14].

In its paper [15] Bar-Yam and colleagues equated the first assumption to an ‘autocratic regime’ in which few master genes govern the entire behavior of the cell. In the same paper, the authors criticize this view for a so-called ‘intermediate’ regime in which both local and global motions of the transcriptome machinery take place in response to a given stimulus. In the local mode, few master genes are in fact maximally affected by a given condition but, given the high connectivity of the gene regulation network, these initially local motions are transformed into a general motion of the entire transcriptome so that the system can acquire a new ‘equilibrium state’ correspondent to the new phenotype and involving the entire set of gene products.

In so doing the authors introduce the concept of ‘energy’, i.e. the need to think of a global minimization principle in order to explain the presence of a relatively small number of cellular phenotypes (cell kinds) with respect to the infinite number of possibilities offered by the different combinations of gene expression levels [15,1].

**Figure 2:**
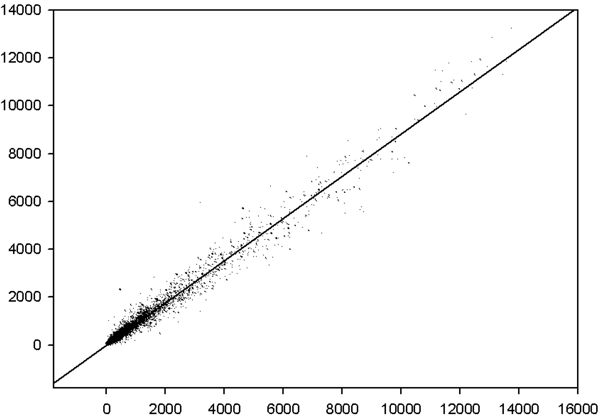
**The picture reports the microarray derived differential gene expression values correspondent to two samples of the same cell kind.** They refer to 22690 ORFs and span four order of magnitudes of expression levels.

On a practical ground, the common experience of any experimentalist dealing with microarray data is the fact that any two independent samples of the same cell kind when correlated over the expression of more or less twenty thousands different gene products display a near to unity correlation (see Figure 2).

This organization, spanning four order of magnitudes of gene expression levels and encompassing tens of thousands of gene products is a very remarkable fact of nature calling for an explanation and clearly supporting the crucial importance of a thorough investigation of its origin by a statistical mechanics perspective [1,2]. We will go back to this theme in the last paragraph of the results section, here we will concentrate on the falsification of the second assumption, i.e. the supposed irrelevance of cell-cell interactions necessary for concentrating only on single cell explanations of population based measures like microarray studies.

When asynchronously (as for reproductive cell cycle) cultured, freely growing colonies of cells are normally considered as ergodic ensembles in which each cell behaves independently of the others, this allows to refer the data coming from population made of billions of cells to a single average cell. If this hypothesis is tenable, we should not observe any relevant temporal structure arising from such cell ensembles, being all the relevant (and well known) cell based rhythmic activities averaged out, at the colony level, by the substantial independence of cells. This was demonstrated not to be the case in a paper by Tsuchyia et al . [14] where different cellular systems, going from yeast, to human fibroblasts were shown to present marked periodicities in time as for their gene expression levels .

**Figure 3:**
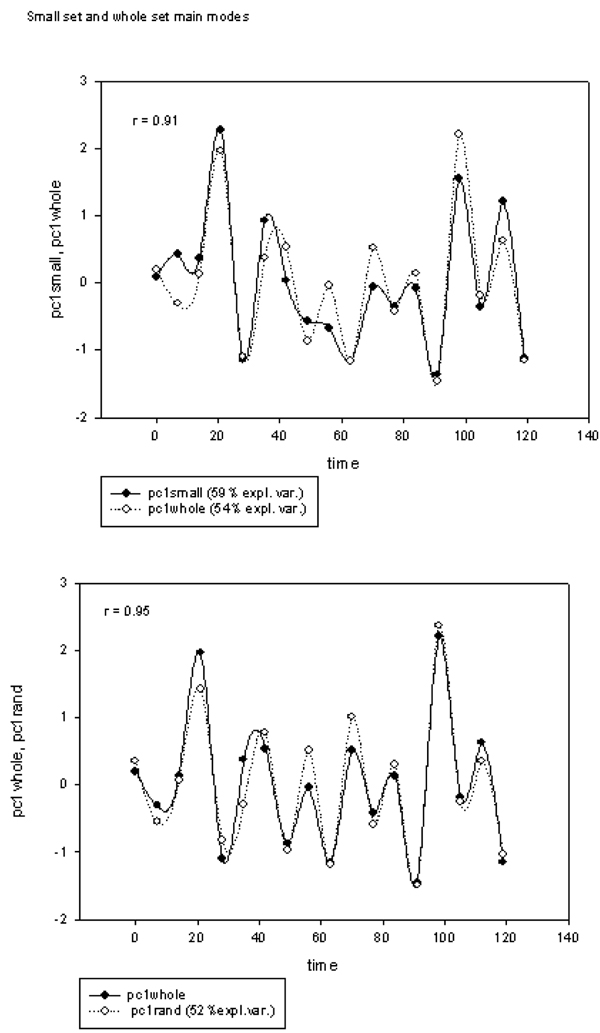
**The first component of the variation in time of different gene choice is reported as for free growing yeast colonies.** Pc1small corresponds to a choice of 60 genes connected to ribogenesis, pc1whole to the entire genome, pc1rand to a random extraction of 275 genes. It is worth noting the superposition of the time courses and the practical invariance of the percent of explained variance of the extracted mode.

Figure 3 reports the first principal component of gene expression as measured in freely growing yeast colonies and computed over different choices of genes. While metabolic cycles in yeast colonies were already very well known and associated to the alternation of ‘reductive’ and ‘oxidative’ phases [16], in this case the relevant feature of the observed cycles was their independence of any specific biological function of the involved gene products. The same time course of the first principal component of gene expression was observed with different choices of gene probes, going from the whole genome, to ribogenesis related genes and random gene extractions. This implies the impossibility to get rid of this phenomenon in terms of specific gene functionalities or cell physiology features and the need to think of a sort of ‘ecology-in-a-plate’ arising from the interaction of many cells living together and interacting each other and thus asking for a different kind of ‘statistics’ with respect to the simple ‘average cell hypothesis’ implicit in the classical molecular paradigm [17]. The observed behavior is analogous to the behavior of paramagnetic substances (e.g spin glasses) which acquire specific macroscopic organization thanks to the existence of preferred orientations of nearby elementary units (e.g. dipoles) [7].

**Figure 4:**
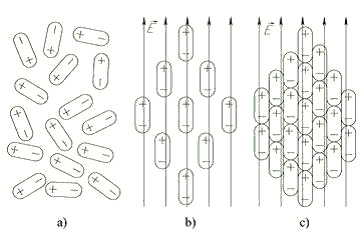
**A simplified schema of a dipole gas is reported:** in the absence of an external field the dipoles only display local order with a statistical tendency of opposite polarities to come close each other (panel a). In panels b) and c) an increasingly powerful external field acts on the system inducing a global order of the dipoles.

This kind of systems (Figure 4) can collectively respond to externally applied stimuli (e.g. electromagnetic fields) acting as order parameters so acquiring information processing capabilities [18].

This is in line with the dynamics toward general ‘attractors’ correspondent to different cell kinds and influencing the entire transcriptome as demonstrated in the case of blood cell differentiation [1,19]. The link between the spin-glass systems and differentiation dynamics is made more cogent by the use of the authors (to demonstrate a collective order spanning the entire transcriptome) of the (GEDI Gene Expression Dynamics Inspector.) algorithm deriving by the so called SOMs (Self Organizing Maps) and implementing a classical statistical mechanics renormalization approach in which each element of a system re-orientates itself coherently with the orientation of the neighbors and iterating the same model at different scales [1,19,20]. This paradigm is potentially extremely useful for biological systems description as already stated by many authors involved in protein folding dynamics [8,21], given it allows for the establishing of different hierarchies of order correspondent to different scales in both space and time [21] and in the meanwhile it offers a rational explanation to the presence of sharp thresholds allowing the system to undergo very different trajectories (and consequently very different final attractors) starting from the same initial configuration [1].

This is the case of the generation of very different cell kinds from the same stem cell population described by Huang [1].

### iii) Innate immune response: local, global and scalable

The last example I will discuss has the goal to summarize three landmarks of the suggested perspective: the relation holding between local (specific genes/proteins) and global (genome-wide/proteome-wide) effects and the cognate concept of scalability, i.e. the possibility to reproduce some general effects by a randomly down-sized version of the entire system [22]. For this goal use is made of the results of a study I participated in the recent past [23,24].

The general setting of the study follows the classical molecular biology approach: three strains of mice, two of them knocked out of a gene known to deeply affect a biological process, while the third is knocked out of both, were selected. The phenotypes (in terms of genome-wide transcriptome) of cultured cells coming from the above mice were compared between them and with the phenotype of a wild-type strain so to dissect the effect of the particular mutations (the knocked-out genes) on the behavior of the other genes involved in the same process.

**Figure 5:**
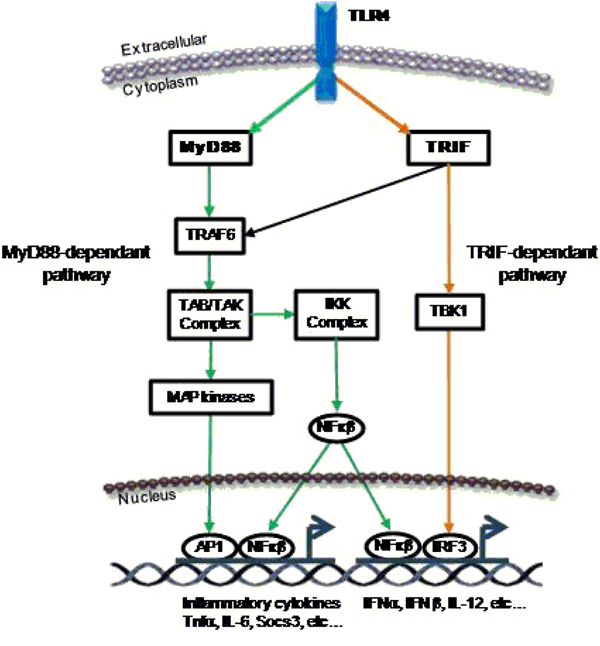
The LPS stimulus processing pathway.

In this case we were dealing with mutations affecting the so called innate immunity process, i.e. the response set for by organisms when invaded by a potentially pathogen biological entity (generally a bacterium). The defense response starts from very specialized cells called macrophages: the innate immune system utilizes pattern-recognition receptors (PRRs), proteins present on the cell membrane that are able to recognize and bind to pathogen associated molecules, such as lypopolisaccharides (LPS). LPS, which are located in the outer membrane of Gram-negative bacteria, after being recognized by the macrophage receptors, trigger a cascade of signaling events eventually leading to the digestion of the bacteria. Two effector proteins that are essential for the correct execution of the above process are called MyD88- and TRIF- (see Figure 5).

It is only after MyD88- and TRIF- (or both) are activated that a number of signaling molecules (cytokines and interferons) are produced [25,26]. These molecules effectively start the so called ‘inflammatory process’ by activating other cell populations (namely helper T-cells) for the onset and correct execution of the entire immune defense process by which foreign intruders are eliminated and immunological memory is created [25,26].

This is an extremely specific mechanism whose activation follows a very precise sequence of events in time, for this reason the elimination of Myd88- or TRIF- genes is expected to partially impair the obtained response, while the contemporary elimination of both Myd88- and TRIF- genes should abolish the acute immune response with no production of cytokines. For this reason this is an almost ideal model to study the contemporary presence of local, acute responses and general, systemic, responses of the gene regulation network that in turn correspond to the global rearrangement of the gene regulation network following the acute response. If the classical ‘purely molecular’ view of gene regulation holds, we expect that macrophages coming from MyD88- and TRIF- knocked out mice, when challenged with LPS stimulation, to have a severe impairment in the expression of genes linked to the immune response, while the other should remain substantially unchanged. In the case of double knock-out mice we expect the expression increase of the genes induced by MyD88- and TRIF- proteins to be completely abolished and, again, all the other genes to maintain the same characteristic level of expression. Clearly the wild-type group is expected to have the immune related genes abruptly up-regulated after the LPS stimulus while maintaining the same basic level of expression for the other genes. This is the classical molecular biology view separating a set of so called house-keeping genes that keep an invariant level of transcription because they are responsible of the maintaining of the cell, i.e. the business-as-usual, and regulated genes whose activity undergoes abrupt changes in expression according to external stimuli. In this view genes are implicitly considered as independent units that are switched on or off by an invisible hand that correctly matches the genes with the cell needs. An alternative view looking at the entire genome like an highly connected dynamical system gives a different prediction: the whole genome expression will ‘sense’ the LPS stimulus (with the only possible exception of double knock-out that in principle has no possibility to sense the incoming stimulus) by globally shifting on another (slightly) different level of expression while the immune related genes neatly increase their expression. The global shift in expression corresponds to the ‘resonance’ on the entire regulation network of the abrupt increase of some specific genes.

We analysed the whole genome expression of Affymetrix standard platform encompassing 22690 different ORFs after LPS stimulation of murine macrophages and refer to 12 experimental conditions (4 genotypes at 3 time points): Wild type, MyD88KO, TRIFKO, and Myd88/TRIF DoubleKO (DKO) at 0 (t0), 1 (t1) and 4 h (t4). The basic metrics adopted for the genotype comparison in terms of entity of response to the LPS stimulus was the Pearson correlation coefficient on the entire genome [22,23] computed on the whole genome expression vector (state vector in Huang terms [1]) as well as on different extractions of specific gene subsets (random and immune-related). Unlike the use of DNA microarrays to identify specific genes we, treated genes as anonymous members of a single ensemble containing N genes and calculated the samples similarity in terms of Pearson correlation. As stated by Chang et al. [19] this ensemble property of the population of genes is a robust measure of the samples similarity not being affected by noise at the level of individual gene similarity. We adopted four different choices of genes extractions as basis for computing autocorrelation with t0 state vector: entire genome, only cytokine, random extraction of genes, and ‘connector’ genes respectively. These choices correspond to different mesoscopic views on the gene regulation system. Global motion of gene regulation network as a connected system is registered by the entire genome basis, while the scalable character and gene-function-independence of this motion is caught by the random extraction choice. The local motion of specific genes responsible for the acute effect of LPS stimulation is registered by the cytokine choice.

The so called ‘connector’ genes refer to a collection of 136 genes selected by means of a principal component analysis that was recognized to discriminate DKO samples from the other ones so pointing to the difference from genotypes correspondent to a systemic immune response observable at the level of the entire organism (wild type, Myd88KO and TRIFKO) and samples coming from DKO mice that do not display any general immune response. These 136 genes list has inside many well known immune related genes as well as other genes involved in the systemic response to immune stimulus like apoptosis related genes.

**Figure 6:**
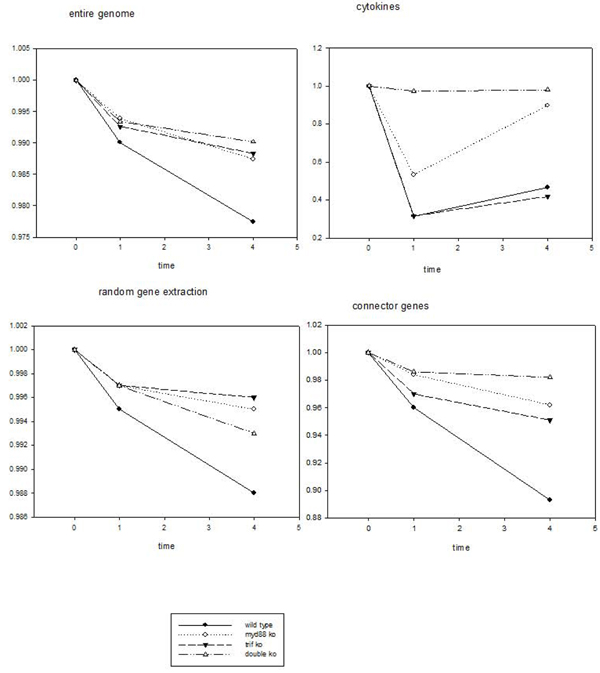
**The figure reports four different gene choices correspondent to different mesoscopic views on the gene regulation system (see text).** The graphs have the Pearson correlation coefficient r with t0 state vector as ordinate and time (hours) as abscissa and report the dynamical response to LPS stimulus for each genotype, a lower value of r corresponds to a more marked response.

Figure 6 reports the autocorrelation distribution in time (Pearson r with the t0 sample) for all the four genotypes relative to the above described choices of genes. In the case of the entire genome (top left panel) we observe a major departure from unit correlation correspondent to a greater response, as expected, in the case of wild type. The three mutated genotypes all displayed a very minor albeit reliable and monotonically related to time departure from unity correlation pointing to the ‘global sensing’ of LPS stimulation. The presence of a strong attractor-like structure constraining the genome-wide expression at the cell population level into a very sharply defined configuration spanning the entire set of gene expression values allows for only minor departures from unit of the autocorrelation in time. Shifting to a random choice of 100 genes (bottom left panel) we can observe exactly the same pattern displayed by the entire genome basis. We iterated many times these random choices observing a completely invariant picture starting from minimal choice of around 60 genes [22]. This is a confirmation of the ‘scalable’ character of gene expression network that constitutes a strongly connected set whose general connectivity can be appreciated starting from a minimum sample of elements.

Top right panel is related to the cytokines local response, it is immediate to note the much bigger displacement from t0 (before LPS stimulus) with respect to the global response, this is in line with the location of cytokines just after the ‘perceived stimulus’ along a sequential pathway. As expected, DKO does not appear to have any response in terms of cytokine expression, consistently with its complete lack of LPS receptors, while the other three genotypes show a marked departure from t0 state vector with a non-linear character (as opposed to the monotonous global motion) reaching its maximum at 1h , Myd88KO was much more seriously affected than TRIFKO as for this acute response.

Connector genes (bottom right panel) display an intermediate behaviour in between acute and collective motion, from collective motion (probably due to their elevated number) they inherit a minimal displacement from unit correlation of DKO, from their link with acute response they inherit both the more marked displacement from unit with respect to aspecific global motion and the expected ranking of detrimental effect of mutations with Myd88 KO more affected than TRIFKO.

The presence of a ‘global response’ encompassing the entire genome expression in the case of DKO is surprising. This response, albeit minimal, it is present and strongly invariant across different choices of gene subsets and point to a possible decoupling between acute and global response of the system implying the presence of an alternative ‘pattern recognition pathway’allowing the DKO cells to sense the stimulus without being able to evoke an acute massive response.

An exacerbated sensitivity to apparently minor stimuli and a general resilience of the entire system stay together side-by side in biological systems. This apparent paradox can be explained by the consideration of biological systems as very strongly interconnected network systems. Some nodes of these networks (in this case those corresponding to cytokines), thanks to their peculiar location in the network architecture, are responsible for the sensitivity aspects, while the large degree of interconnection is at the basis of the resilience properties of the system, here appearing as a global adjustment of the entire system following the stimulus.  All in all these results falsify the simplistic view of autonomus ‘house-keeping’ and ‘regulated’ genes compartments and point to an attractor-like dynamics of global gene expression at the cell population level. Our interpretation goes on the same line of the results obtained by Nykter et al. [28] that analyzed the same biological system (global gene expression data from macrophage cell populations) demonstrating, by means of information theory based methods, the presence of criticality. Criticality [28] is another crucial landmark of self-organized dynamical systems and corresponds to a state of the system in which perturbations are propagated over long temporal or spatial scales. In the case of macrophages this corresponds to the spreading of the initial stimulus to the genome wide expression we observed to be mediated by the connector genes.

## Conclusions

There are some general points we can derive from the consideration of the above described results. First of all the necessity to take into consideration even when, like in the case of yeast metabolic network, we concentrate on a single element (the lethal character of a specific mutation), the general frame in which the element is inserted. This style of reasoning has the name ‘mesoscopic’ [8] because concentrates on the between elements relation structure that is considered as the channel along which the microscopic and macroscopic views are related.

The mesoscopic view implies a statistical approach to the studied system that in turn implies that the same macrostate can be generated by a multiplicity of microstates so strongly limiting the possibility of a one-to-one mapping between a given network configuration and the observed behaviours. Not only the same network architecture (in terms of wiring diagram) can ‘occupy’ a multiplicity of different states (see [1] for a thorough discussion of the difference between network architecture and network states), but different network architectures can give rise, to the same state vector of their elements through different pathways so to be virtually not discriminable starting from the output [27]. The plasticity and state dependence of the kinetic parameters of the same network adds indeterminacy to the picture and asks for a statistical perspective.

This statistical perspective is made cogent by the recognized fact that a largely stochastic and unpredictable behavior at the single cell level is paralleled by very strongly repeatable phenomena observed when a large population of cell is taken into consideration. This blend of microscopic stochasticity and macroscopic determinism is the signature of statistical mechanics and, in general, of condensed matter physics.

Cell biology since its birth relied on population data coming from millions of cells growing on plates but sketched its models by means of node and arrows cartoons implicitly posited in the interior of a single cell. The contemporary discovery of single cell highly noisy and unpredictable behavior and of the presence of collective order parameters spanning four order of magnitudes of expression intensity involving thousands of genes simultaneously at the cell cultures level forces cell biologists to a complete change of paradigm. The complementation of local ‘hard-wired’ explanation of cell biology by means of specific interactions between localized portions of the regulation network and of the global, collective, and independent of specific elements approach is a crucial point in systems biology in order to deal with a big mass of emerging data about collective rhythms involving the entire cellular machinery at population level as well as the delocalized and scalable modification of the entire transcriptome in differentiation processes. The idealization of cell kind as ‘attractors’ of gene expression networks made by some authors [1,17,19,29], is in my opinion extremely fruitful because it encompasses the two basic ingredients of ‘population determinism’ and ‘single cell stochasticity’ we sketched before. The cell populations can shift from an attractor to another thanks to their intrinsic noise correspondent to the fact the cells inside each population are not equal among them and the cells at the ‘extremes’ of a distribution can be the ‘pioneers’ of an attractor shift [1].

The presence of self sustained oscillations in cell ensembles points to a sort of ‘structuring’ of the intercellular variance with the consequent departure of the cell cultures from ergodicity and the need to consider a sort of ‘ecology-in-a-plate’ and higher level constructs linked to cell-cell communication with respect to classical cell-centered molecular biology models [14].

This point is particularly cogent if we take into consideration a second aspect of the ergodic assumption that must be kept distinct from the loss of temporal structure due to lack of synchrony we described in this work: namely the common but unarticulated notion that fluctuations in individuals (single cells), even if synchronized, are too fast so they are not apparent at the usual scale of observation, again we imply the substantial equivalence between all the cells inside a population. This is a very important point because it is strictly related to the onset of non-genetic heterogeneity between cells, the demonstration of a stable between cells heterogeneity in a population (both in terms of gene expression and protein content) should allow for going further in the direction of establishing a parallel between statistical mechanics and biology, being the ‘temperature’ of the system (the cell population) correspondent to the relative occupancy of different microstates (at the basis of the observed heterogeneity) by the single constituting cells [30,31]. In particular, in [31] based on analysis of skewed scale-dependent distributions of gene expression level in diverse eukaryotic cell types, a random and sporadic “basal” transcription mechanism (analog of low-temperature fluctuations) for protein-coding genes in eukaryotic cell types was predicted. Physically, the “basal” transcription of genes might reflect “nonlinear responses” of the independent gene transcription molecular complexes to internal or external fluctuations including thermal molecular motion. Many of the lower level basal transcription events may be essential for determining normal development and pathological cell phenotypes. It was suggested that the “basal” mechanism of gene transcription might enhance the expression of rarely-expressed genes due to noise-driven stochastic resonance regulatory signals and, thus, provide a switch in a basic level of phenotypic diversity, adaptability and random mono-allelic expression in cell populations [31].

The above frame of the references could be able to accommodate in a consistent frame the problem of biological noise that until now had a very questionable and vague status, while in the above envisaged dynamical paradigm it could play an essential role in biological response as discussed in [19] and implicitly predicted for normal and abnormal cells in [31].

This is, in my opinion, an extremely fascinating avenue for a substantial advancement in biological science, as for now, the consideration of biological systems in terms of networks is a first step toward the acquiring of a new perspective on cell biology that will shift this science from a naïve mechanistic perspective to a more broad view encompassing dynamical systems science concepts and findings.

## Competing interests

The author declares that he has no competing interests.
